# Parliament and the Profession

**Published:** 1918-02-02

**Authors:** 


### Parliament and the Profession.

### More Pensioners, Fewer Soldiers, in Voluntary Hospitals.

Mr. Hodge, the Minister of Pensions, informed Sir Henry Harris that arrangements have now been made with both London and provincial hospitals, and the terms will be communicated to all Local War Pensions Committees forthwith. The Minister was fully aware of the difficulty which is being experienced in London in obtaining in-patient treatment for discharged disabled men, largely due to the sudden withdrawal by the War Office of facilities for such treatment in military hospitals. In response to his representations the War Office, recognising the prior claim of the Ministry to the accommodation in civil hospitals, will refrain from requisitioning further beds in these hospitals and, also, will release the accommodation now occupied by them as rapidly as possible. A medical department has been instituted, presided over by a very eminent surgeon, which has the whole matter in hand, and it is hoped that difficulties as regards numbers of men waiting for treatment will rapidly be swept away.

### Military Dental Service.

Mr. Pennefather asked the Under-Secretary of State for War if he had received a copy of a report which a non-party Parliamentary Committee on the relation of military mental service to man-power propose to publish, and, if so, what steps the War Office propose to take in regard to the Committee's recommendations. Mr. Macpherson, in his reply, indicated the views and action of the War Office in this matter. He said that the importance of conservative treatment of soldiers' teeth in this country is fully recognised, qualified dental surgeons and lorries are supplied as demanded by commanders in the field, specially qualified dental surgeons are associated with surgeons in the treatment of jaw cases, dentists and dental machines are being withdrawn from combatant units as this becomes necessary, and there is an inspecting dental surgeon in each command, who supervises the dental work and advises the Deputy-Director of Medical Services.

### R.A.M C. Officers in India.

Mr. Macpherson informed Colonel Yate that steps are being taken to relieve as many as possible of the medical officers who have been a long time in India, but in view of transport and other difficulties it is quite impossible to give a date in connection with this, or even to promise that all such officers will be relieved.

